# Is Accurate N – Staging for Gastric Cancer Possible?

**DOI:** 10.3389/fsurg.2018.00041

**Published:** 2018-05-31

**Authors:** Chrysovalantis Vergadis, Dimitrios Schizas

**Affiliations:** ^1^Department of Radiology, Laikon General Hospital, Athens, Greece; ^2^First Department of Surgery, National and Kapodistrian University of Athens, Laikon General Hospital, Athens, Greece

**Keywords:** N – staging, computerized tomography, magnetic resonance imaging, positron emission tomography, endoscopic ultrasonography, gastric cancer

## Abstract

Node stage (N stage) is of paramount importance for gastric cancer staging. Radiologically node status implies detection and characterization of suspect malignant lymph nodes. Clinically it might determine survival and alter therapeutic plans. A number of modalities, including computerized tomography, MRI, PET and endoscopic ultrasound are currently available. Using a multimodality strategy, accuracy ranges between 50–90% across various studies. Specificity and sensitivity varies with respect to method, number of positive lymph nodes, their location and other characteristics. Restaging after neoadjuvant therapy and staging of recurrence presents its own, particular challenges. Each method has its advantages and limitations and none of them alone is adequate enough for staging. While most of them are clinically well established, they are also active research topics. To overcome the aforementioned limitations a multidisciplinary, multimodality approach with emphasis on clinical staging and treatment plans is proposed.

## Introduction

Gastric cancer is the fourth commoner cancer and the second most common cancer – related death cause (1). Currently surgery is the cornerstone of curative therapy. Unfortunately, with the exception of countries applying population-wide screening programs, most patients will arrive to clinician’s attention with extensive or inoperable disease. For advanced gastric cancer (AGC), neoadjuvant treatment modalities gain significant survival advantage (2). A small subset of patients will present with early gastric cancer (EGC) and for those patients endoscopic treatment, in the form of endoscopic mucosal resection or endoscopic submucosal dissection, is appropriate, sparing morbidity and mortality associated with radical surgery (3). 

Accurate staging is prerequisite for application of different treatment strategies. Staging of gastric cancer follows, as for most solid cancers, TNM staging system (4). N staging is based on number of positive lymph nodes (Table 1) and implies detection of malignant lymph nodes, determination of their number and mapping of their anatomic location. Lymph nodes location is commonly classified according to Japanese Research Society for Gastric Cancer Guidelines: Compartment I includes the perigastric lymph nodes. Compartment II includes lymph nodes along the left gastric artery and common hepatic artery, around the celiac axis, at the splenic hilum, and along the splenic artery. Compartment III includes lymph nodes in the hepatoduodenal ligament, at the posterior aspect of the head of the pancreas, and at the root of the mesentery. Compartment IV includes lymph nodes along the middle colic vessels and the paraaortic lymph nodes.

**Table 1:** AJJC N staging for gastric cancer.

Nx	Regional lymph nodes cannot be assesed
N0	No regional lymph node metastases
N1	Metastasis in 1–2 regional lymph nodes
N2	Metastasis in 3–6 regional lymph nodes
N3	Metastasis in 7 or more regional lymph nodes
N3a	Metastasis in 7–15 regional lymph nodes
N3b	Metastasis in 16 or more regional lymph nodes

While N staging should classify the patient accurately based on the above criteria, it should also be focus on 2 questions: (1) N_0_ vs N_+_ because existence of positive lymph nodes will probably drive the patient to neoadjuvant therapy while non – existence makes him a candidate for minimally invasive endoscopic treatment (2) Existence of distant and malignant lymph nodes that are oncologically equivalent to metastasis and preclude surgical treatment.

After neoadjuvant therapy, disease will be downstaged for a subset of patients. While same principles apply for re-staging as for primary staging, anatomic and physiologic alterations caused by neo-adjuvant treatment should be considered for correct results interpretation. Finally follow – up after radical surgery implies accurate N-staging for assessment of resectable recurrent disease.

Imaging modalities for N staging are computerized tomography (CT), MRI, PET and endoscopic ultrasound imaging (EUS). Those and some newer modalities will be briefly discussed in the following mini review.

## Computerized Tomography

Computerized tomography is the cornerstone of gastric cancer staging (5). MultiDetector row Computerized Tomography (MDCT) allows faster data collection, eliminating breathing motion artifacts and more accurate synchronization with contrast bolus, allowing better differentiation between arterial, portal and venous phases. Detailed study of the enhancement during various phases can provide useful information on their possibility of malignancy (6, 7). Imaging products are images of high resolution, multiplanar imaging and 3 dimensional reconstruction (8, 9). Those modalities have significantly improved T staging, allowing better discrimination between AGC and EGC. Benefits for N staging remain unclear.

Main MDCT criterion for characterizing a lymph node as malignant is size criterion. Threshold of 6 mm of shorter dimension for celiac axis lymph nodes and 8 mm for perigastric lymph nodes is often used arbitrary, with many researchers preferring smaller ones (10). Other criteria in use are round shape, central necrosis, heterogenous enhancement, loss of fatty hilum and clustering of 3 or more lymph nodes (8). However no worldwide consensus exists. Except of size, the other qualitative criteria are applied at the discretion of the radiologist leading to significant intraobserver variability (11). 

Even size criterion is not objective. Lymph nodes infiltration will lead to their enlargement in a non-linear and often unpredictable manner. Microscopic lymph nodes metastases will not cause lymph nodes enlargement, causing false negative results. Lymph nodes might be enlarged due to other causes than malignant infiltration, including inflammatory reaction to primary tumor, consequences of chemotherapy or other, irrelevant to gastric cancer, pathology, causing false positive results (8). An equilibrium between specificity and sensitivity exists, depending on the selected size threshold. In other words choosing a smaller threshold as pathologic will increase sensitivity at the expense of specificity and vice versa (11). More or less other criteria suffer from similar limitations. A combination of criteria for malignancy is commonly used, both in research and clinical practice, but this practice is of unproven value (10). 

Enhancement of lymph nodes during portal phase might be useful in increasing specificity: An enlarged lymph node from other cause than malignancy will not, or will at a lesser extent, enhance. Considering than enhancement is a quantitative rather than a qualitative parameter, level of enhancement can help characterize an already enlarged lymph node in an objective manner, increasing specificity (12). 

A recent meta-analysis by Kwee et al (10) showed that specificity for MDCT ranges between 62.5 and 91.9% while sensitivity varies between 50 and 89.9% across various studies. Due to high variability authors did not proceed to pooling of the results. Notably different criteria used (including different threshold for criterion size) did not significantly change sensitivity, specificity or other parameters of diagnostic accuracy. This underlines that limitations of MDCT in staging are inherent and partly only depend on study parameters. Over staging and under staging are equally distributed. Diagnostic accuracy varies with N stage. In the study by Ohashi et al (13) accuracy was 32.6% (18–47.1%) for N_1_, 53.8% (37.5–70.1%) for N_2_ and 46.2% (32.1–60.2%) for N_3_.

Considering the above, it is not surprising that MDCT is somewhat inaccurate for N – staging. Recent technical developments such as multiplanar imaging and 3D reconstructions significantly improved T staging but did not improve diagnostic accuracy of N - staging (8, 9). 

MDCT lacks accuracy in post-neoadjuvant assessment of gastric cancer. Enlarged lymph nodes due to inflammatory changes caused by chemotherapy and inability to differentiate lymph nodes necrosis due to tumor infiltration or tumor response to treatment blur the commonest diagnostic criteria and constitute inherent limitations. Specificity of MDCT is about 57% while sensitivity about 43% (9). 

## Magnetic Resonance Imaging

Intrinsic ability of MRI to produce soft tissue discrimination is a useful tool in staging of gastric cancer. Until recently technical limitations and limited availability prevented its wide use. Recent technologic developments like 3.0T field scanner, high speed sequences and other, increased spatial resolution and improved soft tissue characterization (8, 14). Certain sequences, particularly Diffusion Weighted Imaging (DWI), allows in – depth imaging interpretation with possible clinical benefits (15). Furthermore new contrast agents with affinity for nodal tissue are an active research topic in literature. The above have renewed interest in MRI staging for gastric cancer.

Main MDCT criteria for characterizing lymph nodes as malignant are also used for MRI: Size criterion, contrast enhancement, central necrosis, loss of fatty hilum and lymph nodes clustering have been used in research with variable results. A recent meta – analysis compared MRI performance in discriminating N_0_ vs N_+_ disease. The pooled accuracy of MRI to diagnose N stage was 0.78 (95% CI, 0.72–0.83). Summary estimates of sensitivity, specificity, positive likelihood ration and negative likelihood ration were 0.86 (95% CI, 0.80–0.92), 0.67 (95% CI, 0.54–0.79), 0.21 (95% CI, 0.13–0.33) and 12.75 (95% CI, 6.31–25.77), respectively (14). Compared with MDCT, MRI presents similar diagnostic accuracy, specificity and sensitivity (15). Compared with PET, MRI presents better specificity but inferior sensitivity (16). While most studies suffer from limitations such as small sample size, heterogeneity in used technique, non prospective character or measurement in different samples, they also establish MRI role in gastric cancer staging, particularly N staging.

Re evaluation of DWI sequence has evoked new, interesting data on N – staging. Briefly DWI is capable of quantifying diffusion and perfusion phenomena in a tissue. Malignant infiltration causes disruption of normal architecture, increased tissue cellularity and fibrosis altering cellular density. Consequently extracellular space is decreased, limiting perfusion phenomena. Those alterations in cellular density are reflected in DWI sequence, especially in cases of subcentimeter infiltrated lymph nodes (17). Primary data (15) indicate that MRI with DWI shows better diagnostic accuracy than MRI without DWI for N staging (specificity: 86.7 vs 50%, sensitivity: 58.8% vs 90%). Other studies have reproduced those results, indicating better diagnostic accuracy for MRI with DWI, although this is not always constant or statistically significant (8, 18)

Ultrasmall superparamagnetic iron oxide (USPIO), a contrast agent with affinity for reticuloendothelial tissue has potential interest for N – staging. Tissue infiltration by malignancy alters lymph node topography. Different contents produce different magnetic signal intensity in certain MRI sequences, leading to field heterogeneity (19). This technique is currently under active investigation but its clinical importance has not been clarified.

## Positron Emission Tomography

18F-fluoro-2-deoxy-D-glucose (FDG) is a radiolabelled glucose analogue that accumulates in cells through increased glucose uptake. Glucose transporters overexpress in neoplastic cells so to serve their increased metabolic needs, leading to accumulations of radiotracer and their subsequent detection. Radiologic result is cumulative and analogous to tumor burden in a certain anatomic location alongside with extent of expression of glucose transporters (20). 

PET scan is of limited accuracy for N1 staging due to relatively low spatial resolution that does not allow discrimination between primary tumor and locoregional lymph nodes. Sensitivity for N1 disease is low ranging from 18–46% (20) Furthermore PET scan has a lower tracer accumulation for diffuse and mucinous cancer types, both common in gastric cancer, and associated with a worse prognosis (21). According to a recent meta – analysis PET sensitivity for N – staging is 52% and specificity is 88% (16). PET accuracy remains low as N-stage increases, with sensitivity ranging between 33–46% for N2 and 44–63% for N3 disease (20). Specificity instead is high for PET scan as it can reach 91–100% (22) A PET scan can alter treatment plans as lymph nodes outside the operation field are considered oncologically equivalent to metastasis and can render the disease unresectable or drive the patient to definite chemotherapy or palliative treatment (22). Validity of PET/CT is also limited for peritoneal disease, as peritoneal implants present low metabolic activity. Rate of occult peritoneal disease after PET scan ranges between 20–35% across the literature (23). 

Combination of PET scan and CT scan significantly improves sensitivity and specificity. PET/CT has higher accuracy in preoperative staging (68%) in comparison to PET alone or CT alone and thus should be preferred (4). 

Assessment of tumor response to neoadjuvant therapy is usually estimated by tumor size reduction in terms of volumetry or major dimension. Another possible index is reduction of glucose uptake in PET/CT. Fractional change in glucose uptake is instant after one circle only of chemotherapy and can be used for estimating histopathologic response and even overall survival (24). Those principles also apply to N staging.

Another use of PET/CT is detecting tumor recurrence. Patterns of recurrence after curative gastric cancer surgery include intraluminal recurrence, nodal basin recurrence and distant metastases. Follow up after gastric cancer surgery is usually performed through CT and endoscopy. Unfortunately those methods are structural and their interpretation is often confused by normal post-operative changes. Ultimately follow – up strategies are not superior to clinical surveillance and do not gain survival benefits (25). While PET/CT does not seem valuable as single follow up modality due to relative high rate of false – negative results (21), it is useful for interpreting equivocal findings after CT. In this scenario sensitivity and specificity are high, ranging between 80 and 95% across various studies (26). Furthermore, PET/CT results can often alter treatment plans for as many as 50% of the patients (26). 

## Endoscopic UltraSound

EUS technique for N staging is same as for T staging: With the patient in left lateral position, a radial or linear probe is inserted in the stomach endoscopically. Frequencies used vary between 5 and 20 MHz with 7.5 MHz being the frequency most commonly used. During slow withdrawal from the pylorus to esophagogastric junction the echoendoscope is used to assess the whole perigastric area for lymph nodes. Other locoregional areas such as mediastinal lymph nodes, celiac axes lymph nodes and cervical lymph nodes can also be assessed, with various sensitivity and specificity across various studies (27). Endoscopic criteria for suspect lymph nodes are size of the node, round shape, echopoorness, sharp demarcation of surrounding fat and nodes at the vicinity of the tumor. Importantly, none of the above criteria alone presents high specificity, sensitivity of prognostic value. A combination of the above criteria alongside with data derived from other imaging modalities and clinical experience produces best results (28). 

 Main utility of EUS lies with determination T staging, with sensitivity and specificity ranging >90% in high quality studies (29). Results for N staging are somewhat inferior with accuracy ranging between 50 and 80% (9). A recent Cochrane database metanalysis summed up previous research and proved that sensitivity is 83% (CL: 79–87%), specificity 67% (CL: 61–72%), positive likelihood ration 2.5 (CI: 2.1–2.9%), negative likelihood ration 0.25 (CI: 0.20–0.31) and diagnostic odds ratio 10 (CI: 7–13) (29). 

Reasons limiting accuracy of EUS for N staging are (1) limited penetration. With frequencies used, penetration depth is 1–6 cm (30). Nodes further than that distance will not be recognized (2) limited accuracy of EUS in certain areas of the stomach, including prepyloric region, angle of the stomach and gastroesophageal junction (31). (3) the fact that none of the used criteria alone is sufficient for characterizing a lymph node malignant. (4) EUS is an operator – dependent examination with results varying widely between different operators and use of different equipment. (5) Inability of EUS probes to pass through stenotic areas (e.g., Esophagogastric junction) (27, 32) Utility of EUS FNA is higher for N_1_ staging and declines as N – Staging increases, with sensitivity and specificity ranging between 60–70% for N_2–3_ tumors

Addition of fine needle aspiration (FNA) at suspect lymph nodes significantly increases diagnostic accuracy of EUS. Sensitivity is approximately 92%, specificity 98% and positive prognostic value 98% (9). However this method also has its limitation: Not all suspect lymph nodes can be examined due to tumor interference (possible contamination/spread), interference of vital structures or non visualization (32). Other means for increasing EUS FNA specificity is considering that positive lymph nodes are more likely in ACG. However characterizing a tumor as N_0_ is critical for Τ_is_ or T_1_ as this establishes criteria for endoscopic treatment. Finally marking of suspect lymph nodes that will later be harvested during surgery has potential implications in guiding lymph – nodes clearance (33). 

Utility of EUS for detecting distant metastatic disease is poor as limited penetration depth does not allow examination of distant structures. A probable application however is detection of previously unsuspected ascites: Sensitivity is probably higher than CT or US examination and might predict unresectable disease (34). 

In summary, usefulness of EUS FNA in N staging of gastric cancer is not pivotal, as it is for Τ staging, but remains an important adjunct to other staging techniques. For EGC a negative N staging is necessary for establishing applicability for minimally invasive techniques. In these cases, vigorous examination of suspect lymph nodes is important as positive N staging precludes endoscopic treatment. EUS role is limited in AGC cancer: Other staging techniques will usually provide necessary information. Its role in confirming non respectability is also limited, with the exception of confirming direct infiltration to non – resectable structures or confirmation of ascites.

## Conclusions

Considering the above, N-staging for gastric cancer is challenging and seems to lack accuracy. Each and all diagnostic modalities have limited sensitivity and specificity due to inherent limitations. (Table 2) CT, MRI, PET-CT and EUS can’t consistently detect lymph node involvement in gastric cancer, even in the era of advanced technology. When the efficacy of MDCT is taken as a reference, DWI represented a complementary imaging technique, while 18F-FDG PET/CT had limited utility for preoperative N-staging except in cases of ambiguous, distant lymph nodes. Combined with MDCT, EUS-FNA increases diagnostic accuracy and establishes N_0_ characterization status. No single modality alone can provide all necessary information. However accuracy for the sake of accuracy might not answer to clinical questions. As stated before, discrimination between N stages does not have equal clinical importance. For example an accurate discrimination between N_0_ and N_+_ will alter radically therapeutic decisions while discrimination between N_1_ and N_2_ to a lesser extent.

**Table 2:** Comparison between different diagnostic modalities.

	Accuracy	Specificity	Sensitivity	Indication	Limitation
MDCT	40–50%	60–90%	50–90%	Basic staging study	Lacks accuracy in neo- adjuvant and follow – up setting
MRI	70–80%	70–90%	55–75%	Similar benefits to MDCTNewer modalities (DWI sequence, newer contrast agents) have the potential to increase accuracy	Not widely availableSome of its characteristics are still under investigationExpensive
EUS	50–80%	80–90%	70–80%	Accurate for regional lymph nodesAddition of FNA offers tissue confirmation	Inadequate for distal metastasesOperator – dependentInherent limitations in certain anatomic areas
PET	30–40%	40–50%	>90%	High sensitivityDetects distant metastases with higher sensitivity than other modalities	Inadequate for loco – regional diseaseHigh rate of false positive resultsExpensiveNot widely available

Considering the above, a rational diagnosti protocol should start with MDCT that could classify patient in N_0_ or N_+_ status. A N_0_ patient should undergo EUS, to confirm his N status. If patient’s status is N_+_ and distant lymphadenopathy is detected, he should undergo PET – CT for further characterization of his disease. MRI should be used if MDCT is contradicted or for characterization of ambiguous findings or for research purpose

A multimodal and active approach to clinical questions seems the best option. Multiple approaches, depending on proposed therapeutic plan should investigate the N status of the patient. Radiologist, as a part of a multidisciplinary team, should maintain an active role in proposing, performing and interpreting imaging results. Furthermore limitations of imaging should be known to other members of the aforementioned team. Treatment plans must take the above limitations in consideration so to ensure best treatment options and patients’ best interests.

## Author Contributions

CV performed literature search, contributed to final text and had scientific supervision. DS performed literature search, data mining and drafted the manuscript.

## Conflict of Interest Statement

The authors declare that the research was conducted in the absence of any commercial or financial relationships that could be construed as a potential conflict of interest.
